# WIRELESS PH MONITORING AND CONVENTIONAL ESOPHAGEAL PH MONITORING: COMPARATIVE STUDY OF DISCOMFORT, LIMITATIONS IN DAILY ACTIVITIES AND COMPLICATIONS

**DOI:** 10.1590/0102-672020210001e1566

**Published:** 2021-05-14

**Authors:** Rimon Sobhi AZZAM, Gabriela Barge AZZAM, Ary NASI

**Affiliations:** 1 Hospital das Clínicas, Department of Gastroenterology, Faculty of Medicine, University of São Paulo, São Paulo, SP, Brazil; 2 Faculty of Medicine, University of Santo Amaro, São Paulo, SP, Brazil

**Keywords:** Gastroesophageal reflux, Esophageal pH monitoring, Wireless technology, Refluxo gastroesofágico, Monitoramento do pH esofágico, Tecnologia sem fio

## Abstract

**Background:**

The catheter of the esophageal pH monitoring is associated with nasal and throat discomfort, and different behave in patients. The capsule of the wireless pH monitoring may cause chest pain and complications.

**Aim:**

To compare the wireless and conventional pH monitoring concerning the degree of discomfort and limitations in daily activities, complications, ability to diagnose pathological reflux, and costs.

Methods: Twenty-five patients with symptoms of gastroesophageal reflux were prospectively submitted, in a simultaneous initial period, to 24-hour catheter esophageal pH monitoring and 48-hour wireless system. After removing each system, patients underwent a specific clinical questionnaire.

**Results:**

Fifteen patients (60%) pointed a higher discomfort in the introduction of the capsule (p=0.327). Discomfort and limitations in daily activities were lower on 2^nd^ day (p<0.05); however, continued to be expressive (32% to 44%). Chest pain occurred in 13 (52%) patients. The diagnostic gain of pathological reflux was 12% with the wireless system (p=0.355).

**Conclusions:**

1) There is no significant difference between the discomfort mentioned in the introduction of the capsule and the catheter; 2) during reflux monitoring, the wireless system provides significant less discomfort and limitations in daily activities; 3) there is no significant difference between the two methods in the ability to diagnose pathological reflux; 4) wireless pH monitoring has higher cost.

## RESUMO

**Racional:**

O cateter da pHmetria esofágica associa-se ao desconforto nasal e na garganta, e comportamento diferente nos pacientes. A cápsula da pHmetria sem cateter pode causar dor torácica e complicações.

**Objetivo:**

Comparar as pHmetrias sem cateter e a convencional, em relação ao desconforto e limitações das atividades diárias, complicações, capacidade de diagnosticar refluxo patológico, e custos.

**Métodos:**

Vinte e cinco pacientes com sintomas de refluxo gastroesofágico foram prospectivamente submetidos, em um período inicial simultâneo, à pHmetria esofágica com cateter durante 24 h e à pHmetria sem cateter durante 48 h. Após a retirada de cada método, pacientes responderam o questionário clínico específico.

**Resultados:**

Quinze pacientes (60%) relataram maior desconforto na introdução da cápsula (p=0,327). Desconforto e limitações das atividades diárias foram menores no 2º dia (p< 0,05); entretanto, continuaram sendo expressivos (32% a 44%). Dor torácica ocorreu em 13 (52%) pacientes. O ganho diagnóstico no refluxo patológico foi de 12% com o sistema sem cateter (p=0,355).

**Conclusões:**

1) Não há diferença significativa entre o desconforto relatado na introdução da cápsula e do cateter; 2) durante a monitorização do refluxo, o sistema sem cateter proporciona significativo menor desconforto e limitações das atividades diárias; 3) não há diferença significativa entre os dois métodos na capacidade de diagnosticar o refluxo patológico; 4) pHmetria sem cateter tem custo maior.

## INTRODUCTION

Esophageal pH monitoring was introduced into clinical practice in in patients in the 70’s^15^. Flexible catheters and portable pH recorders began to be used in outpatients in the early 80’s. The prolonged (18 to 24 h) pH monitoring of the distal esophagus allowed a quantitative measure of gastroesophageal reflux and a better understanding of the gastroesophageal reflux disease (GERD)^5^ ^,^ ^6^ ^,^ ^15^.

An international Consensus Group developed a global definition of GERD as “the condition which develops when the reflux of stomach contents causes troublesome symptoms and/or complications”^29^. The classic symptoms of GERD are heartburn (burning feeling in the retrosternal area) and regurgitation (perception of refluxed gastric contents into the mouth or pharynx)^26^.

Upper gastrointestinal endoscopy and esophageal pH monitoring are the two methods directly related to GERD diagnosis^21^. The first method identifies the disease forms causing esophagitis, while the second diagnoses pathological gastroesophageal reflux^21^. Endoscopy also allows the collection of biopsy material for histological study, and such procedure is of fundamental importance in the study of the GERD complications^21^.

Esophageal pH monitoring is considered the gold standard for the diagnosis of pathological gastroesophageal acid reflux. The conventional method (with catheter) has sensitivity ranging from 79% to 96%, specificity 85% to 100%, and 98% accuracy^8^ ^,^ ^10^ ^,^ ^14^ ^,^ ^16^ ^,^ ^19^ ^,^ ^27^ ^,^ ^30^. The wireless method (without catheter and with capsule) has similar sensitivity and specificity (78.3% to 100% and 84.5% to 94.8%, respectively)^23^.

In clinical approach, the esophageal pH monitoring has precise indications and provides interesting details of the gastroesophageal acid reflux: assesses presence and intensity of acid reflux, characterizes reflux pattern (orthostatic, supine or combined), and associates clinical complaint with acid reflux episodes^21^.

However, during the monitored period, the conventional catheter is associated with nasal and throat discomfort and patients tend to exhibit reduced food intake and behave differently^8^ ^,^ ^20^. Although the wireless system has been developed to avoid restrictions and improve the diagnostic sensitivity, it’s capsule may cause chest pain in up to 65% of cases^1,4,23, 24,28,34^.

This study was motivated by no local and few international publications concerning the comparative study of the discomfort and limitations in daily activities between wireless pH monitoring and conventional esophageal pH monitoring^3^.

The present study aimed to compare both esophageal pH monitoring, with and without catheter, concerning the degree of discomfort and restrictions in routine activities, complications, ability to diagnose pathological gastroesophageal reflux, and costs.

## METHODS

This study was approved by the Ethics Committee for Analysis of Research Projects of the Clinical Hospital of the São Paulo University Medical School (number 1079/06).

Patients referred to the Esophageal Functional Investigation Laboratory of the Digestive System Surgery Department of the Clinical Hospital of the São Paulo University Medical School, São Paulo, Brazil, were prospectively screened for esophageal pH monitoring.

Inclusion criteria were: heartburn and/or regurgitation as the main clinical complaint; at least 18 years of age; recent upper gastrointestinal endoscopy (within the last two months); interruption in the administration of proton pump inhibitors for seven days preceding the pH monitoring; and signature of the free and informed consent form. Exclusion criteria were: esophageal diverticula, strictures and varices; hiatal hernia greater than or equal to 3 cm; erosive esophagitis with Los Angeles C or D grades; Barrett’s esophagus; and neoplasms, obstructive diseases or previous surgery of the gastrointestinal tract.

All patients underwent clinical interview, upper gastrointestinal endoscopy, nasal and oral esophageal manometry, pH monitoring with and without catheter (for 24 and 48 h, respectively, with simultaneous initial period) and a specific clinical questionnaire of discomfort and limitations in daily activities.

The following GERD complaints were investigated during clinical interview: typical (heartburn and regurgitation), esophageal atypical (chest pain and globus sensation), and extraesophageal atypical (cough, asthma, dysphonia and hem).

All patients underwent endoscopy at the Gastrointestinal Endoscopy Department of the Clinical Hospital of the São Paulo University Medical School. The presence of erosive esophagitis and hiatal hernia were assessed. The Los Angeles grade system was used for the characterization of esophagitis; and the protrusion of part of the stomach 2 cm or more above the diaphragm, during deep inspiration, was considered hiatal hernia.

Before esophageal pH monitoring, a conventional esophageal manometry was performed to locate the lower esophageal sphincter (LES) for positioning the pH sensors.

### Esophageal pH monitoring

After evaluating the distance of the LES, in relation to the nostril and to the upper dental arch through esophageal manometry, a catheter of the conventional esophageal pH monitoring was introduced, followed by the capsule of the wireless esophageal pH monitoring. Each patient underwent, with simultaneous monitoring recording starting time, conventional pH monitoring for 24 hours, and wireless pH monitoring for 48 h.

The equipment used for the conventional pH monitoring consisted of portable pH recording device (Medtronic/Synectics, USA), pH calibration solutions and pH monitoring flexible catheter (Alacer, Brazil). The 2.1 mm in diameter catheter displayed two antimony sensors (2 cm away from each other) for pH registration and an external reference electrode. The catheter sensors were systematically calibrated before each test, using the calibration solutions at pH 7.0 and pH 1.0. The distal sensor was positioned 3 cm above the superior border of the LES, which was identified through nasal esophageal manometry, to monitorate the reflux at a more distal level. The proximal sensor was positioned 5 cm above the superior border of the LES to monitorate the reflux at the internationally accepted standard position.

The wireless pH monitoring equipment (Bravo, Medtronic/Synectics, USA) consisted of portable pH recording device, pH calibration solutions, pH monitoring capsule and capsule delivery device. The pH monitoring capsule, measuring 6.0x6.3x26.0 mm, contained one antimony sensor, sensitive to pH changes, and an internal reference electrode. The capsule sensor was systematically calibrated before each test, using the same calibration solutions at pH 7.0 and pH 1.0. The capsule was inserted through the mouth, with the assistance of the capsule delivery device that does not require endoscopy, and positioned in the esophagus, 3 cm above the superior border of the LES at the same level as the distal sensor of the conventional catheter. The suction system was applied by a vacuum pump (510 mmHg during 60 s) and the esophagus mucosa penetrated into the capsule compartment (4 mm in diameter). The pin was released, transfixing the suctioned mucosa, while attaching the capsule to the esophageal wall. The vacuum was turned off and the capsule released from the distal end of the delivery device, which was removed. The pH recording was started and transmitted by radio waves (telemetry) to the portable recording device.

Patients were advised to try to maintain their daily activities, to fill out the pH monitoring log, and to return to the laboratory: after 24 h (1^st^ day) to remove conventional pH monitoring system (catheter and external recording device); and again after another 24 h (2^nd^ day) to remove the external recording device of the wireless pH monitoring system.

It is noteworthy that parameters of normality for the characterization of pathological reflux were established by measuring reflux 5 cm above the LES and were only used in this study as reference values. The normal parameters used were: rate of total reflux time up to 4.5%, rate of reflux time in an upright position up to 8.4%, and rate of reflux time in a supine position up to 3.5%^14^.

The patient was considered to be affected by pathological gastroesophageal reflux if any of the three percentages of reflux time adopted were at levels higher than normal; or had quantitatively normal reflux, but with a significant relationship with the symptoms. The relationship between clinical complaint and gastroesophageal acid reflux was assessed by the Symptom Index and considered positive when equal or greater than 50%^33^.

### Questionnaire of discomfort and limitations in daily activities

The patients also underwent a specific clinical questionnaire after removing each type of pH monitoring (1^st^ and 2^nd^ day), to compare the degree of discomfort and limitations in daily activities of the two types of esophageal pH monitoring. The questionnaire about the degree of discomfort and limitations in the daily activities was idealized by the authors, as long as there is no validated international questionnaire for this purpose. Parameters were considered. Initially, it was requested the patient to answer about the catheter and the capsule which bothered more to be introduced?

To evaluate the degree of discomfort and limitations in the daily activities during the first day of monitoring (in which the patient was simultaneously with the conventional catheter and the wireless capsule), in comparison with the second day (patient with the capsule, but without the catheter), it has been searched the interference in the routine activities, nasal discomfort, runny nose, cervical discomfort, feeding alteration, sleep disturbance, concern with the equipment, discomfort without bath and social constraint for the appearance of the equipment. For each item, the patient was requested to choose a number on a scale from zero to ten in accordance with the degree of discomfort. Zero was equivalent to the absence of discomfort and ten was an intense one. The discomfort degree was grouped in three categories: mild (score from 1 to 3), moderate (4 to 6) and intense (7 to 10).

Finally, for the following items of the questionnaire, it was requested the patient to answer yes or not: Did you leave house? Did you work? Did you have chest or epigastric pain? Would you repeat wireless pH monitoring if needed? and Would you repeat the conventional pH monitoring if needed?

### Statistical analysis

For the statistical study, conducted at the Laboratory of Statistics and Epidemiology, Department of Gastroenterology, Clinical Hospital, São Paulo University Medical School, the following tests were used: bilateral proportion test, Friedman test, and unilateral proportion test. Descriptive level of p<0.05 was considered significant.

## RESULTS

Twenty-five patients meeting the inclusion criteria were enrolled in the study and submitted to the two types of esophageal pH monitoring (with and without catheter). Twenty-one (84%) of which were females and the age ranged from 34 to 73 years (average 52.4). The clinical complaints were predominantly typical of GERD in all patients, esophageal atypical in 16 (64%) and extraesophageal atypical in 19 (76%). Upper gastrointestinal endoscopy revealed erosive esophagitis in 8 (32%) patients and hiatal hernia in 11 (44%).

Patients pointed a higher degree of discomfort in the introduction of both types of pH monitoring. Fifteen (60%) pointed discomfort in the introduction of the capsule while 10 (40%) with the catheter. Although most cases of discomfort occurred with the introduction of the capsule, such difference did not reached levels of statistical significance (p=0,327).

In terms of the questions formulated in the specific clinical questionnaire, there was significant reduction in the degree of discomfort and limitations in the daily activities on the 2^nd^ day compared to the 1^st^ day on all the analyzed items (p< 0,05, Table 1). However, in 2^nd^ day, an expressive contingent of patients still presented interference in the routine activities (36%), cervical discomfort (32%), feeding alteration (44%), sleep disturbance (32%) and concern with the equipment (44%, Table 1).

**TABLE 1:** Description and comparison of the discomfort degree on the 1st and 2nd day

Degree of discomfort	Day	Absence n (%)	Mild n (%)	Moderate n (%)	Intense n (%)	TOTAL n	p^1^
Interference in routine activities	1^st^ day 2^nd^ day	3 (12,0) 16 (64,0)	9 (36,0) 7 (28,0)	8 (32,0) 2 (8,0)	5 (20,0) 0 (0,0)	25 25	0,001*
Nasal discomfort	1^st^ day 2^nd^ day	5 (20,0) 22 (88,0)	9 (36,0) 1 (4,0)	3 (12,0) 1 (4,0)	8 (32,0) 1 (4,0)	25 25	0,002*
Runny nose	1^st^ day 2^nd^ day	5 (20,0) 19 (76,0)	10 (40,0) 4 (16,0)	6 (24,0) 1 (4,0)	4 (16,0) 1 (4,0)	25 25	0,011*
Cervical discomfort	1^st^ day 2^nd^ day	0 (0,0) 17 (68,0)	12 (48,0) 2 (8,0)	5 (20,0) 6 (24,0)	8 (32,0) 0 (0,0)	25 25	0,001*
Feeding alteration	1^st^ day 2^nd^ day	9 (36,0) 14 (56,0)	5 (20,0) 5 (20,0)	6 (24,0) 5 (20,0)	5 (20,0) 1 (4,0)	25 25	0,011*
Sleep disturbance	1^st^ day 2^nd^ day	11 (44,0) 17 (68,0)	4 (16,0) 6 (24,0)	2 (8,0) 2 (8,0)	8 (32,0) 0 (0,0)	25 25	0,003*
Concern with the equipment	1^st^ day 2^nd^ day	6 (24,0) 14 (56,0)	6 (24,0) 5 (20,0)	5 (20,0) 4 (16,0)	8 (32,0) 2 (8,0)	25 25	0,003*
Discomfort without bath	1^st^ day 2^nd^ day	2 (8,0) 25 (100,0)	5 (20,0) 0 (0,0)	3 (12,0) 0 (0,0)	15 (60,0) 0 (0,0)	25 25	0,000*
Social constraint	1^st^ day 2^nd^ day	13 (52,0) 23 (92,0)	5 (20,0) 1 (4,0)	1 (4,0) 1 (4,0)	6 (24,0) 0 (0,0)	25 25	0,008*

^1^ Friedman test

Eleven (44.0%) have left home in the 1^st^ day and 17 (68.0%) in the 2^nd^ day of monitorization. Nine (36.0%) have worked in 1^st^ day and 22 (88.0%) in the 2^nd^ day of monitorization. It was observed that a large number of patients left home and worked in the 2^nd^ day of monitoring in relation to the 1^st^ day (leave home: p=0,044; work: p<0.001). However, in the 2^nd^ day, an expressive contingent of patients (32%) did not leave home.

Thirteen (52%) patients presented chest or epigastric pain during the total period of monitorization.

When patients were inquired if there was a need for repetition of the examinations, 24 (96.0%) affirmed that they would repeat the conventional pH monitoring and 22 (88.0%) would repeat the wireless pH monitoring. It did not have a significant difference between the two types of pH monitoring due to the patient’s decision to repeat the examination, in case of necessity (p=0,297).

Pathological gastroesophageal reflux was detected by the conventional method in 16 (64%) patients and by the wireless method in 19 (76%). However, this 12% increase in the diagnostic gain has no statistical difference (p=0.355).

With regard to complications, the early capsule drop has occurred in one (4%) patient during the wireless method exam and there was no relevant technical failure in the group monitored with catheter. There was no significant difference between the two types of pH monitoring concerning technical failure during examination (p=0.463). No patient experienced severe chest pain or any other symptom requiring endoscopic removal of the capsule. On the 30^th^ day after the capsule insertion, the spontaneous detachment of the capsule from the esophageal wall was confirmed in all patients, in the study by a lateral chest X-ray. Esophageal perforation, migration, aspiration or other complications have not occurred in any case.

About the expenses, the capsule (single use) costs $411.53 and the catheter (reused for five times) $39.22; so, the catheter costs only $7.84 per use.

## DISCUSSION

This is the first Brazilian study that compares the esophageal pH monitoring with and without catheter regarding discomfort, limitations in daily activities, complications and costs. However, the small number of patients is a limitation of this study; this restriction occurred due to the cost of the capsules. 

It was observed that 68.0% of the cases submitted to endoscopy did not have erosive esophagitis; in this group of patients, esophageal pH monitoring was indicated for diagnosis or exclusion of non-erosive GERD. In 32.0% with erosive esophagitis, pH monitoring was indicated to characterize the reflux pattern.

Classical contraindications described in the literature of the wireless pH monitoring system are important limitations to the method and include: severe esophagitis, esophageal varices, bleeding diathesis, anticoagulation, stricture or obstruction of the gastrointestinal tract, and the use of cardiac pacemaker or defibrillator^25^. It should be noted that such conditions do not represent contraindications to the conventional pH monitoring with catheter. The impossibility of properly fixing the capsule in an area of esophagitis, with intense degree of inflammatory process, prevents the evaluation of pH monitoring reflux in an important group of patients with GERD. The capsule is released spontaneously after a few days and is eliminated by the digestive tract; however, the presence of stenosis or obstruction of the gastrointestinal tract would cause capsule impaction.

Exclusion criteria for this study included esophageal diverticula due to the risk of perforation during the capsule introduction. It should be emphasized that the wireless pH monitoring may have limited use in patients under investigation of non-cardiac chest pain, because the capsule can cause chest pain, making it difficult to discern pain caused by the capsule rather than reflux or heart. The capsule contains a small magnet; therefore, a Magnetic Resonance is not recommended within 30 days after insertion of the capsule due to the risk of perforation if the capsule has not been completely eliminated. These restrictions also do not apply to conventional pH monitoring with catheter.

Possible contraindications to wireless system should be carefully evaluated before the test and if they are noted, a conventional pH monitoring with catheter should be performed^25^. Patients should also be informed about the risks of wireless pH monitoring: discomfort, chest pain, dysphagia, odynophagia, foreign body sensation, nausea, vomiting, laryngospasm, vasovagal reaction, capsule fixation failure, premature capsule detachment, failure of the capsule detachment, capsule migration, capsule aspiration, capsule retention, esophageal tear or ulcer, bleeding, perforation and possible endoscopic or surgical procedure to solve complications.

The comparative study between wireless and conventional esophageal pH monitoring was conducted at a lower traditional level of the esophagus. Reasons for this choice included: changes in the mucosa due to GERD commonly occur next to the esophagogastric junction; feasibility study of inserting the capsule closest to this transition zone; and, at a lower level, compare discomfort, activity restrictions, complications and pathological gastroesophageal reflux detection between the two types of pH monitoring sensors (capsule vs. catheter).

The present and Andrews et al.^2^ studies observed that there was no significant difference between the capsule or catheter introduction. However, Gillies et al.^11^ showed less discomfort in the introduction of the capsule (p<0.0001).

Studying catheter interference in daily activities of patients only submitted to conventional esophageal pH monitoring, Fass et al.^8^ demonstrated a significant reduction in duration of activities (patient tends to be more sedentary on exam day), number of meals, and frequency of reflux symptoms during monitoring.

By comparing discomfort and interference in routine activities between wireless and conventional pH test, during the monitoring, Andrews et al., Gillies et al., and Wong et al.^2^ ^,^ ^11^ ^,^ ^34^ observed better tolerability of the capsule. There was significantly less discomfort (nasal pain, runny nose, cervical pain, cervical discomfort and headache), as well as less interference in daily activities (general activities, eating, work and sleep)^2^ ^,^ ^11^ ^,^ ^34^. However, Andrews et al.^2^ showed more thoracic discomfort in the wireless pH monitoring than in the conventional pH monitoring (p=0.001).

Maerten et al.^18^ describe that the main inconvenience of wireless pH monitoring is the induction of thoracic discomfort, which may range from mild foreign body sensation to intense thoracic pain, resulting from the capsule fixation on the esophageal wall. In the present study, thoracic or epigastric pain was observed during monitoring in 52% of patients. This finding is consistent with the literature, which shows chest pain from 10.5% to 65% of patients undergoing wireless pH monitoring^1^ ^,^ ^4^ ^,^ ^23^ ^,^ ^24^ ^,^ ^28^ ^,^ ^34^. Studying the presence of symptoms related to the capsule, Remes-Troche et al.^11^ observed: chest pain (in 33% of cases), foreign body sensation (14%), nausea (6%) and more than one symptom in 11% of the cases.

The finding of 4% of early drop of the capsule during wireless pH monitoring supports literature data, indicating that this technical failure occurred in 4.1% to 5% of cases^4^ ^,^ ^11^ ^,^ ^24^.

With chest radiological control on the 14^th^ day, Lin et al.^17^ observed that the capsule remained in 1% of cases. There are reports of endoscopic capsule removal in 1.4% to 3.5% of cases^1^ ^,^ ^4^ and the most common reason for withdrawal was severe chest pain^23^.

Other rare complications of the wireless pH monitoring reported in the literature are: esophageal perforation during insertion, esophageal ulcer, capsule dislodgement in the pyriform sinus, capsule migration to the nasopharynx after cough, aspiration of the capsule into the lower lobe bronchus, and capsule retention in a colon diverticulum^7^ ^,^ ^9^ ^,^ ^12^ ^,^ ^13^ ^,^ ^31^ ^,^ ^32^. Because of this complication, we believe that a simple abdominal x-ray should also be required for complete evaluation of the capsule elimination.

The present study effectively proved that, during monitoring, the wireless pH monitoring provides a significant reduction in the degree of discomfort and limitations in daily activities; however, it was evidenced that the presence of the capsule was associated with chest pain in an expressive number of patients. Moreover, it proved that better tolerability does not provide a significant increase in the diagnostic sensitivity of GERD; this fact is corroborated by the literature review published by Maerten et al^18^.

Wireless pH monitoring is a high cost procedure, limiting its use in daily practice, and it has no ability to identify non-acid reflux. Impedance-pH monitoring is a promising method that detects several types of reflux (acid, non-acid, liquid, gaseous), evaluates other important measures (esophagus ability to transport the bolus, basal mucosal impedance and post-reflux primary peristalsis) and is consolidated as a new gold standard for the diagnosis of gastroesophageal reflux^21^ ^,^ ^22^.

## CONCLUSIONS

It can be concluded that: 1) there is no significant difference between the discomfort in the introduction of the wireless pH monitoring capsule and the pH monitoring catheter; 2) during reflux monitoring, wireless pH test provides significant less discomfort and limitations in daily activities compared to conventional pH monitoring; 3) despite the better tolerability of the capsule, there is no significant difference between the two pH monitoring methods in the ability to diagnose pathological gastroesophageal reflux; 4) wireless pH monitoring has higher cost.
